# The Intertwined Roles of Oxidative Stress and Endoplasmic Reticulum Stress in Glaucoma

**DOI:** 10.3390/antiox11050886

**Published:** 2022-04-29

**Authors:** Daire John Hurley, Caoimhe Normile, Mustapha Irnaten, Colm O’Brien

**Affiliations:** Department of Ophthalmology, Mater Misericordiae University Hospital, Eccles Street, D07 R2WY Dublin, Ireland; mirnaten2014@gmail.com

**Keywords:** glaucoma, oxidative stress, reactive oxygen species, endoplasmic reticulum stress, novel therapies

## Abstract

Glaucoma is the leading cause of irreversible blindness worldwide, and the burden of the disease continues to grow as the global population ages. Currently, the only treatment option is to lower intraocular pressure. A better understanding of glaucoma pathogenesis will help us to develop novel therapeutic options. Oxidative stress has been implicated in the pathogenesis of many diseases. Oxidative stress occurs when there is an imbalance in redox homeostasis, with reactive oxygen species producing processes overcoming anti-oxidant defensive processes. Oxidative stress works in a synergistic fashion with endoplasmic reticulum stress, to drive glaucomatous damage to trabecular meshwork, retinal ganglion cells and the optic nerve head. We discuss the oxidative stress and endoplasmic reticulum stress pathways and their connections including their key intermediary, calcium. We highlight therapeutic options aimed at disrupting these pathways and discuss their potential role in glaucoma treatment.

## 1. Introduction

Glaucoma, an age-dependent disease which is more common in the elderly population, is a common cause of visual impairment, with a prevalence of 3.5% in those over the age of 40 and 9% in those over 80 [1,2]. It is the leading cause of irreversible blindness worldwide, with an estimated 79.6 million people suffering the effects of glaucoma in 2020, a number which is expected to increase to 111 million by 2040 [3]. It can be defined as an irreversible and progressive optic neuropathy [4]. Glaucoma is associated with visual field loss, primarily from damage to retinal ganglion cell (RGC) axons as they exit the eye via the optic nerve head (ONH). Glaucoma may be classified as either open, in which the anterior chamber angle remains open, or closed, whereby the aqueous outflow is impaired due to appositional closure of the drainage angle. Both forms may be primary or secondary processes. While the majority of patients with glaucoma have an elevated intraocular pressure (IOP), there are patients with glaucomatous optic neuropathy whose pressures lie within the normal range (normal tension glaucoma), as well as patients whose pressures are higher than normal values, yet have no damage to their ONH (ocular hypertension) [5,6]. These exceptions to the rule would suggest that raised IOP is not the only factor contributing to ONH damage; however, current therapeutic options in the management of glaucoma revolve around IOP reduction either via reduced aqueous production or increased outflow. A better understanding of the molecular pathogenesis of glaucoma will allow for the development of novel therapeutic options in the future.

Oxidative stress occurs when there is an excess of reactive oxygen species (ROS), the partially reduced metabolites of oxygen molecules [7]. These are primarily derived from electron leak in the mitochondrial electron transport chain [8]. This surpasses the buffering effect of the body’s anti-oxidative capacity, comprised of enzymes and nutrients [9]. Oxidative stress is a harmful state that leads to DNA, protein and lipid damage in addition to mitochondrial dysfunction which induces further ROS generation in a vicious cycle [10]. Oxidative stress is heavily implicated and studied in glaucoma at multiple levels and contributes to pro-fibrotic remodelling, IOP dysregulation and impeded RGC axoplasmic transport [10].

The endoplasmic reticulum (ER) is an organelle with a wide variety of functions—it is critical to protein folding and transportation, as well as functioning as a sensor of unfolded or misfolded proteins, which it retains within the ER for eventual degradation [11,12]. Its myriad other roles include lipid synthesis, steroid synthesis, carbohydrate metabolism and drug metabolism [13]. The ER is, in addition, a calcium store, with a balance of intraluminal calcium being key to proper functioning [14]. Normal ER function can also be perturbed by changes in glycosylation, nutrient deprivation, oxidative stress, and hypoxia, any of which can lead to the aggregation of misfolded and unfolded proteins in the ER, a condition known as ER stress [15]. ER stress triggers signalling cascades in a bid to restore homeostasis and a return to normal functioning. These include the less well-known ER overload response (EOR), which promotes proliferation and inflammation [16]. The better understood cascade is that of the unfolded protein response (UPR). The UPR coordinates an extensive response to decrease protein translation, increase protein folding and upregulate protein degradation [17]. It can also direct misfolded and unfolded proteins through the ubiquitin proteasome system, also called ER associated degradation (ERAD) [18]. As well as increasing apoptosis and general autophagy, the UPR also induces a selective form of autophagy, namely, ‘ER-phagy’—the removal of damaged ER [19].

**Figure 1:**
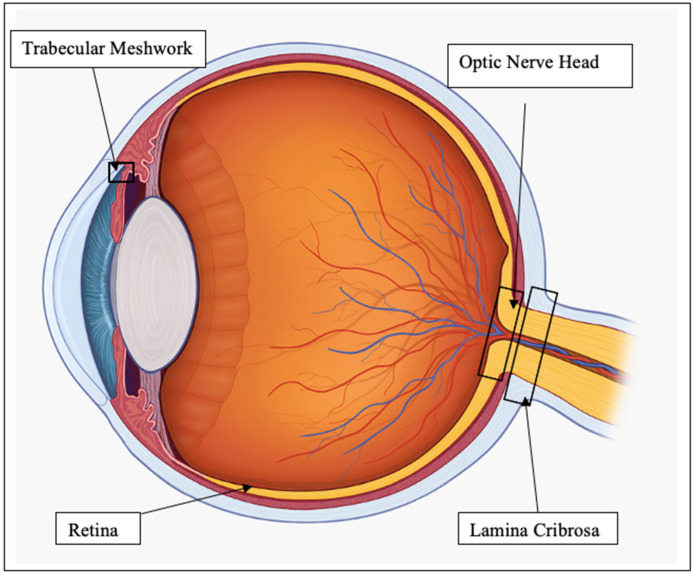
Diagram of eye regions of interest.

The many links between oxidative stress and ER stress are yet to be fully understood. In the ER, redox homeostasis is critical for correct protein folding [20]. Several oxidants can pathologically initiate the UPR, such as ketocholesterol, but its effect can be reversed by treatment with N-acetyl-cysteine, an antioxidant [21,22]. It has been proposed that redox based amplification loops contribute to the switch between adaptive and apoptotic UPR [23]. To discuss in detail the effect of oxidative and ER stress on the eye, we will focus on the regions of the eye most relevant to glaucoma, namely, the trabecular meshwork (TM), RGC, ONH and lamina cribrosa (LC), as shown in Figure 1.

## 2. Oxidative Stress

Oxidative stress occurs when there is an imbalance in redox homeostasis, with pro-oxidative processes that generate reactive oxygen species (ROS) overcoming intrinsic anti-oxidant defence mechanisms [7]. ROS are the partially reduced metabolites of oxygen molecules [9]. They are a subset of free radicals which are substances with unpaired electrons [24].

### 2.1. Pro-Oxidants

ROS may derive from endogenous or exogenous sources. The primary endogenous source of ROS is generated as a by-product in the electron transport chain, involved in aerobic respiration in the mitochondria [25]. This process, known as oxidative phosphorylation (OXPHOS), involves the transfer of electrons through protein complexes (C1–5) coupled to the pumping of hydrogen ions across the mitochondrial inner membrane [26]. This generates a proton gradient that is utilised by ATP synthase to generate ATP [26]. Oxygen serves as the terminal electron acceptor in this pathway, and it binds to hydrogen ions and is reduced to H_2_O as a by-product [8]; however, electron leakage occurs, primarily at the flavin mononucleotide group of complex I (NADH-ubiquinone oxidoreductase) or the ubiquinone site of complex III (ubiquinone-cytochrome c oxidoreductase), and this may reduce oxygen to generate superoxide (O_2_^−^), a potent ROS [27]. This in turn can generate hydrogen peroxide (H_2_O_2_), hydroxyl radicals (OH) and other ROS. Mitochondrial dysfunction results in an excessive release of ROS [28].

Other endogenous sources of ROS include endoplasmic reticulum stress, nitric oxide synthase reaction [29], Fenton reaction [30], polynuclear cells involved in pro-inflammatory responses [31] and the cytochrome p450 system [32]. Exogenous sources that increase ROS production include ionizing and ultraviolet radiation [33], pollutants [34], toxins (including alcohol and smoking) [35], intense physical exercise [36] and polymicrobial infections [37].

### 2.2. Anti-Oxidants

**Figure 2:**
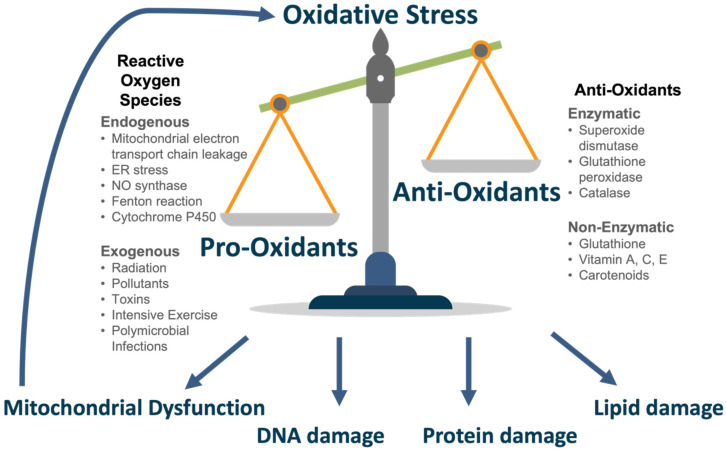
Oxidative Stress.

The body possesses an anti-oxidant defence system to an appropriate level of ROS in normal conditions. This is achieved primarily by enzymes. Superoxide dismutase (SOD) is involved in the conversion of hydrogen peroxide to oxygen (oxidation) and hydrogen peroxide (reduction) [38]. Glutathione peroxidase (GPx) reduces hydrogen peroxide to water or lipid hydroperoxides to their corresponding alcohols [39]. It achieves this using reduced glutathione (GSH), the most abundant anti-oxidant, as a co-factor [39,40]. Catalase is involved in degrading hydrogen peroxide to water and oxygen [41]. Further anti-oxidants include glutathione, ascorbic acid (Vitamin C), α-tocopherol (Vitamin E) and carotenoids which may be synthesised or consumed through diet [42]. In conditions of oxidative stress, the anti-oxidant capacity becomes overwhelmed and insufficient to buffer ROS levels. The delicate balance of pro- and anti-oxidants is seen in Figure 2.

### 2.3. Effects of ROS

ROS act as a direct cytotoxic stimulus, in a caspase dependent or independent manner [43]. They can induce apoptosis [44], programmed cell death or autophagy [45], and lysosomal degradation of a cell’s own constituents. They may induce potent secondary oxidants, such as peroxynitrite, formed from the interaction of superoxide radicals with nitric oxide [46]. ROS may also act as a 2nd messenger, modulating protein function. These oxidative protein modulations increase neuronal susceptibility to damage and result in glial dysfunction in neurodegenerative conditions [47,48]. Another mechanism of ROS damage, of particular concern in neurodegenerative disease, is the accumulation of advanced glycation end products (AGEs). AGEs are non-degradable, detergent insoluble and protease-resistant aggregates that impair tissue function leading to fibrosis [49] and protein function resulting in faulty gene transcription [50]. ROS may stimulate antigen presenting cells (APCs) and activate an autoimmune or inflammatory response [51]. Oxidative stress acts as a deleterious cyclical process with ROS impairing mitochondrial electron transport resulting in further ROS production and oxidative damage [10].

Excessive ROS cause oxidative damage to DNA, proteins and lipids and these may serve as markers for oxidative stress, for example, 8′-hydroxy-2′-deoxyguanosine (8-OHdG) is a DNA oxidative marker [52], while lipid peroxidation is identified by malondialdehyde (MDA) [53]. Finally, Nitrotyrosine, a nitrated amino acid that is formed by peroxynitrite, is a marker of protein oxidative damage [54].

## 3. Oxidative Stress in Glaucoma

Oxidative stress is found in a vast array of pathologies. It is a key feature in neurodegenerative conditions, such as Alzheimer’s [55] and Parkinson’s [56]. Axons in the central nervous system (CNS) are highly susceptible to oxidative damage owing to their high metabolic needs, high lipid and glutamate content and limited regenerative abilities [57]. It is also involved in a number of ocular conditions, such as keratoconus [58], diabetic retinopathy [59], age-related macular degeneration [60] and non-glaucomatous optic neuropathies [61]. It is heavily implicated in primary open angle glaucoma (POAG), a neurodegenerative condition affecting RGC axons. We will examine the role and evidence of oxidative stress in POAG at different ocular tissue levels and systemically.

### 3.1. Trabecular Meshwork

The TM, as the site of aqueous drainage in the anterior chamber, is a key regulator of intra-ocular pressure. Oxidative stress in TM cells contributes to increased aqueous outflow resistance and resultant elevation in IOP. TM cells are the most ROS-sensitive cells of the anterior chamber as they are not sensitised to direct UV radiation [62]. Evidence of oxidative stress is seen in human glaucomatous TM cells with multiple studies showing raised 8-OHdG and a positive correlation with worsening visual fields [63,64]. Nitrotyrosine, a marker for peroxynitrite-mediated injury, shows higher immunoreactivity in TM cells of POAG and this correlates with increased levels of IOP [65]. Peroxynitrite, formed in conditions of oxidative stress, depletes the bioactivity of NO, a free radical that normally relaxes the TM to promote aqueous humour drainage [66]. Reduced anti-oxidant capacity in TM cells is related to progression of POAG [67], and as with all aspects of oxidative stress, this a detrimental cyclical process with excess ROS contributing to elevated IOP which then results in further induction of oxidative free radicals.

ROS promote a pro-fibrotic remodelling of glaucomatous TM tissue [68]. Excessive oxidative stress decreases the function of miRNA-29b, which normally acts to reduce the expression of extra-cellular matrix (ECM) genes [69]. This reduced miRNA-29b function may act to promote ECM deposition in the TM and physically impede aqueous drainage. An accumulation of AGEs has been shown to increase the markers of oxidative stress in human TM cells [70]. They showed that this results in cellular senescence which would lead to further stiffening of the TM cytoskeleton.

Studies have shown evidence of mitochondrial dysfunction in human glaucomatous TM cells [71,72]. He et al. found evidence that mitochondrial complex 1 defects in human glaucomatous TM cells induce ROS release and lead to decreased ATP levels [72]. A study by He et al. found increased levels of calcium (Ca^2+^) in glaucomatous TM cells [71]. This induces the mitochondrial permeability transition pore (MPTP) opening resulting in mitochondrial dysfunction and ROS production.

### 3.2. Optic Nerve Head and Lamina Cribrosa

Oxidative stress at the LC, a mesh-like connective tissue structure at the optic nerve head through which the RGC axons pass, contributes to impeded axoplasmic transport, reduced ocular perfusion and visual field loss. Our lab has previously shown evidence of oxidative stress in human glaucomatous LC (GLC) fibroblast cells in vitro [73]. We found a significant increase in MDA and reduced anti-oxidants, aldo-keto reductase family 1 member C1 (AKR1C1) and glutamate—cysteine ligase catalytic subunit (GCLC), in GLC cells. In the same study, we showed evidence of mitochondrial dysfunction and raised calcium levels in GLC cells [73]. We found a mechano-sensitive pathway of calcium release in LC cells whereby cell membrane stretching, representative of raised IOP, resulted in excessive Ca^2+^ release which may have detrimental effects on mitochondrial functioning [73].

Chidlow et al. found that induced ocular hypertension increased the formation of superoxide radicals and up-regulated ROS-generating NADPH oxidase in ONH cells and RGCs in murine models [74]. ROS stimulates pathological glial activity in the ONH which results in secondary apoptosis of RGCs. This occurs through major histocompatibility complex (MHC) class II upregulation with resultant increased secretion of tumour necrosis factor-α (TNF-α) [75], in addition to activation of the AGE/RAGE (AGE receptors) signalling pathway [57]. There is evidence of oxidative stress in the ONH of POAG patients with higher nitrotyrosine immunoreactivity being noted in the vasculature and glia of the pre-laminar optic nerve head in glaucomatous patients [76]. Untreated glaucomatous ONH astrocytes also exhibit depleted levels of anti-oxidants, such as glutathione [77]. Human ONH astrocytes also display decreased mitochondrial fission and volume density [78] and elevated intracellular cyclic adenosine 3′,5′-monophosphate (cAMP) [79], which promotes further oxidative stress and axonal damage.

The accumulation of AGEs in the LC and vasculature surrounding the ONH increases tissue rigidity and impairs microcirculation [80]. ROS also impact ocular hemodynamics through NO scavenging. This removes a potent vasodilator and has been shown to increase vascular tone in porcine posterior ciliary arteries [81]. This environment makes RGC axons more susceptible to ischaemic damage caused by increased IOP in glaucoma [57]. Excessive ECM deposition in the ONH also stimulates metalloproteinases (MMPs). These proteolytic enzymes contribute to optic nerve head remodelling and may account for the appearance of optic disc cupping [82].

### 3.3. Retinal Ganglion Cells

Oxidative stress leads to apoptosis when ROS production chronically surpasses antioxidant capacity. In glaucoma, RGC apoptosis clinically manifests as optic nerve atrophy and a corresponding visual field loss. Mitochondrial dysfunction and subsequent oxidative stress have been shown to play a key role in RGC death in glaucoma [83,84,85,86]. Mittag et al. found that RGC death occurred after three to four months of elevated intraocular pressure with the surrounding areas of focal loss showing an 18% reduction in mitochondrial membrane potential [83]. RGC death may occur in a caspase-dependent [87] or independent [84] manner during the conditions of oxidative stress. Elevated levels of calcium, indicative of altered mitochondrial bioenergetics, have been shown in pressure-induced RGC apoptosis [88].

Glutamate is the most abundant excitatory neurotransmitter in the human central nervous system [89]. Excessive glutamate levels have been shown to induce oxidative stress and are implicated in numerous neurodegenerative conditions [90]. Vorwerk et al. showed that intravitreal injections of glutamate in rats resulted in apoptosis of RGCs [91]. They subsequently showed further evidence of this relationship as they found that the inhibition of glutamate transporters also leads to RGC death [92]. Glutamate-induced oxidative stress in RGCs is of particular importance as acute elevations of IOP have been shown to elevate glutamate levels [93]. The glutamate analog, N-methyl-D-aspartate (NMDA), has been used to induce oxidative stress in mice leading to raised calcium and ROS levels and ultimately RGC death [94,95]. Studies have also shown that H202, a potent ROS, leads to apoptosis of RGCs through the induction of nuclear factor-kappa B (NF-κB) [96,97].

### 3.4. Other Ocular Tissue and Systemic Oxidative Stress in Glaucoma

Patients with POAG are highly susceptible to oxidative damage as their aqueous and systemic anti-oxidant capacity is 60–70% lower than normal [98,99]. Studies have shown lower serum levels of antioxidant markers, total antioxidant status (TAS) and biologic antioxidant potential (BAP), in patients with POAG [99,100,101,102]. This correlates with higher intraocular pressures [99], higher cup-to-disc ratios [100], worse visual field (VF) loss [101] and lower numbers of RGCs [102]. Total oxidant status (TOS), a marker of oxidative stress, has also been shown to be raised in the serum of POAG patients [103]. Additionally, raised plasma markers of DNA (8-OHdG) [104] and lipid (MDA) [103] oxidation are seen. Serum levels of glutathione show a higher ratio of the oxidised form compared to its usual reduced form [105]. This lower redox index correlated with worse VF loss. Finally, systemic oxidative stress levels have been found to be significantly higher in African-American individuals compared to Caucasians [106,107] and this mirrors their 5–6× increased risk of glaucoma [108].

Research on oxidative stress in aqueous humor samples has yielded similar results to serum samples indicating that local redox status closely mirrors that of systemic antioxidant capacity. A study by Nucci et al. found raised aqueous humor levels of MDA and reduced levels of total antioxidant capacity (TAC) in patients with POAG [109]. In this study, POAG patients also showed evidence of mitochondrial dysfunction with significantly less ATP production versus normal. Interestingly, patients on IOP lowering medications have been shown to have higher aqueous humor levels of protective TAS compared to those not on medication [110]. In an effort to combat oxidative stress, SOD and GPx activity have been shown to be increased in aqueous samples of POAG patients [98,111,112]; however, their protective capacity can be overwhelmed and we see a cumulative rise in ROS as indicated by raised aqueous levels of 8-OHdG and a reduced expression of base excision repair (BER) markers, poly (ADP-ribose) polymerase 1 (PARP1) and oxoguanine DNA glycosylase 1 (OGG1) [113]. Aqueous markers of oxidative stress are also seen in pseudoexfoliation (PXF) glaucoma as demonstrated by the large body of work from Ursula Schlötzer-Schrehardt, [114,115]. She has found greatly reduced levels of ascorbic acid, the most effective free radical scavenger in the eye, in addition to raised markers of oxidative stress in patients with PXF glaucoma, suggestive of a faulty antioxidative defence system [114].

## 4. Endoplasmic Reticulum Stress

### 4.1. Overview

ER stress occurs when the capacity of the ER to correctly fold proteins is saturated. This stress sets off various signal transduction pathways in a bid to either return the ER to its normal equilibrium or to induce apoptosis [116]. Three of these pathways include the unfolded protein response (UPR), the ER overload response (EOR) and the ER associated degradation (ERAD). Three transmembrane ER resident proteins govern three signalling pathways in the UPR, and these are inositol requiring enzyme 1 (IRE1), activating transcription factor 6 (ATF6) and pancreatic ER kinase-like ER kinase (PERK). All three have ER lumen domains which sense ER stress, coupled to cytosolic effector domains. In a normally functioning ER, the ER lumen domains of IRE1, ATF6 and PERK are bound by glucose regulating protein 78 (GRP78) [117]. In a stressed ER, GRP 78 is titrated away, activating the UPR. The primary goal of these pathways is to restore ER protein homeostasis and ensure cell survival, but in the face of persistent activation from unmitigated ER stress, a signalling switch will occur, favouring apoptosis.

### 4.2. IRE1

The branch of the UPR which is most conserved, and most extensively investigated, is that of IRE1. First identified as a component of the UPR in yeast in 1993, there are actually 2 IRE1 genes in mammals—IRE1α and IRE1β [118]. IRE1α is ubiquitously expressed, while IRE1 β has only been found in respiratory and intestinal epithelial cells [119,120]. IRE1 is a type 1 transmembrane protein with three domains—an N-terminal domain in the ER lumen, and an endoribonuclease domain and serine/threonine kinase domain, both in the cytosol [121]. Once activated by ER stress, the luminal domain dimerises and trans- autophosphorylates, leading to the activation of its cytosolic domains [122]. Activated IRE1α excises a 26 base intron from an mRNA that encodes X-box-binding protein 1 (XBP1), which, due to this translational frameshift, becomes the potent transcription factor spliced XBP1 (sXBP1) [123]. sXBP1 upregulates the genes involved in ER protein folding and ERAD [124]. The endoribonuclease domain of activated IRE1α also degrades several ER-localised mRNAs, an activity known as regulated IRE1-dependent decay or RIDD [125]. The activated kinase domain interacts with TNF receptor associated factor 2 (TRAF2) to eventually activate the JNK and NF-κB pathways, integrating ER stress with pro–inflammatory responses [126,127]. Finally, sustained IRE1 activation causes the decay of several microRNAs which eventually leads to activation of the NLRP3 (the NACHT, LRR and PYD domains containing-3) inflammasome [128,129].

### 4.3. ATF6

A type II transmembrane glycoprotein and member of the basic leucine zipper (bZIP) transcription factor family, ATF6 also has two mammalian genes: ATF6α and ATF6β, with the former a potent transcriptional activator, and the latter a poor transcriptional activator [130,131]. Deletion of both causes early embryonic lethality [132]. ATF6 has a C-terminal domain which is in the ER lumen, and an N-terminal domain in the cytosol [133]. In response to ER stress, GRP78 release of the luminal domain allows the transport of ATF6 to the Golgi, where ATF6 is cleaved by site-1 protease (S1P) and site-2 protease (S2P) [134,135]. This releases the cytosolic domain of ATF6 (which contains the bZIP domain), which migrates to the nucleus. In the nucleus it interacts with the ER stress response element (ERSE1) and the ATF/cAMP response element (CRE) to activate numerous target genes, including GRP78 and XBP1, and some components of ERAD [136,137].

### 4.4. PERK

**Figure 3:**
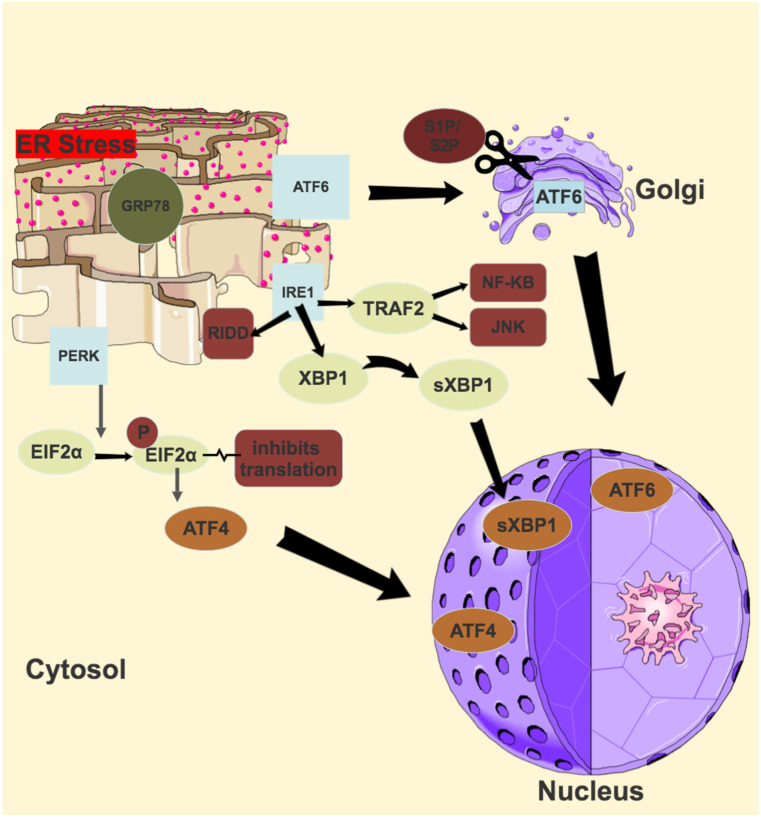
Unfolded protein response (UPR) Pathways.

The final ER stress sensor, PERK, is a type 1 transmembrane protein with an N-terminal luminal domain and a C-terminal serine/threonine kinase domain in the cytosol [138]. In response to ER stress, similarly to IRE1, PERK dimerises and trans-autophosphorylates [138]. Activated PERK phosphorylates eIF2α at Serine5, and this inhibits eIF2β, which normally recycles the eIF2 complex to its active form [139]. Lower levels of this active complex result in lower rates of translation initiation of most mRNA, and a reduced protein synthesis, thereby reducing the protein-folding burden of the ER [140]. Phosphorylated eIF2α selectively translates several mRNAs, including the key transcription factor activating transcription factor 4 (ATF4) [141]. Growth arrest and DNA damage-inducible 34 (GADD34) and transcription factor C/EBP homologous protein (CHOP) are two key genes activated by ATF4 [142,143]. GADD34 is a regulatory targeting subunit of protein phosphatase PPP1, which counteracts PERK by dephosphorylating eIF2α, thereby restoring protein synthesis once the acute phase of ER stress has passed [144]. CHOP, in the setting of chronic or overwhelming ER stress, is a transcription factor which promotes apoptosis [145]. An illustration of the UPR pathways is shown in Figure 3.

### 4.5. Endoplasmic Reticulum Overload Response + Oxidative Stress

Although much less is understood about the EOR than the UPR, it is an important link between ER stress and oxidative stress. The EOR is activated by the several stimuli that also activate the UPR, but the common signal is the accumulation of protein in the ER [16]. Calcium is then released from the ER and subsequently ROS are produced, both of which then result in the activation of nuclear factor kappa-light-chain-enhancer of activated B-cells (NF-κB) [20].

NF-κB is a transcription factor which is key to cell proliferation and inflammation. In comparison to the UPR, the EOR is also more specialised in detecting calcium perturbations in the ER. Transmembrane and Coiled-Coil Domains 1 (TMCO1), encodes for an ER transmembrane protein that functions as a calcium load-activated calcium channel and, in the event of ROS overproduction, can perturb calcium equilibrium and activate the EOR [146].

### 4.6. Endoplasmic Reticulum Associated Protein Degradation

The ERAD is a pathway that targets misfolded proteins in the ER, which, once selected for ERAD, are retro-translocated to the cytosol, where they undergo ubiquitination and subsequent degradation by the proteasome [147,148]. It is implicated in several protein folding diseases, as if ERAD efficiency is compromised or overwhelmed, misfolded proteins will aggregate unless targeted for autophagy. Myocilin, a key gene in glaucoma, when mutated can overwhelm the ERAD and build up in the TM. Glucose regulated protein 94 (GRP94) triages mutant myocilin to ERAD, and studies of GRP94 deletion in mice have shown that subsequent shunting of myocilin from ERAD to autophagy causes less protein aggregation in the TM suggesting autophagy is a more robust pathway to deal with misfolded proteins [149].

## 5. ER Stress in Glaucoma

### 5.1. Overview

**Figure 4:**
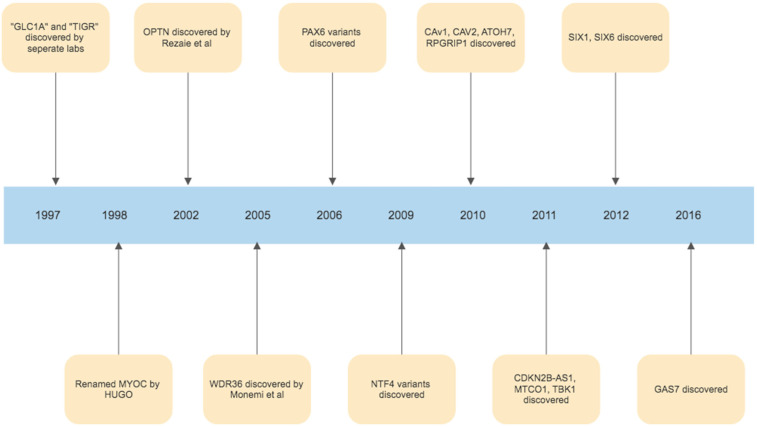
Timeline of gene discoveries linked to the development of glaucoma.

The foundations of our understanding of the role of ER stress in glaucoma were laid in 1997, with the discovery of the myocilin (MYOC) gene. The initial discovery was that an area of chromosome 1q was harbouring a gene associated with juvenile onset POAG, and this area was initially called GLC1A (picked to denote glaucoma, POAG and the fact that this was the first gene linkage site found in glaucoma) [150]. In the same year, it was then realised, that Polansky et al. had previously found, sequenced and named the gene trabecular meshwork inducible glucocorticoid response or TIGR, during studies of corticosteroid induced glaucoma [151,152]. Simultaneously, Kubota et al. in Japan had been studying retinal photoreceptors, and finding a protein similar to myosin, calling it myocilin [153]. In 1998, the Human Genome Organization stepped in and chose the name MYOC, by which the gene has been referred to as since [154]. While MYOC remains the best understood and most investigated gene linked to glaucoma, in the years since many other gene linkages have been identified. (Figure 3). The second gene identified was Optineurin (OPTN), by Rezaie et al., which is a variant with rare frequency but with a high effect size correlated with glaucoma pathogenesis [155,156]. Other similarly rare variants with high effect size in the development of POAG include WD Repeat Domain 36 (WDR36), TANK Binding Kinase 1(TBK1), Neurotrophin 4 (NTF4) and Paired Box 6 (PAX6) [157,158,159,160]. Variants of genes identified in the pathogenesis of glaucoma which are common but have a low effect size in the development of glaucoma include TMCO1, Cyclin-dependent kinase inhibitor 2B (CDKN2B-AS1 or ANRIL), Caveolin 1 (CAV1), Caveolin 2 (CAV2), Sine Oculis Homeobox Homolog 1 (SIX1), Sine Oculis Homeobox Homolog 6 (SIX6), Growth Arrest Specific Protein 7 (GAS7), Atonal BHLH Transcription Factor 7 (ATOH7) and RPGR Interacting Protein 1 (RPGRIP1) [161,162,163,164,165,166,167,168,169]. Identifying these genes not only allows us to potentially target these pathways using novel treatments, but in the future may also allow us to create DNA based tests that could identify those at risk of developing glaucoma, and potentially assist in evaluating who is at a higher risk of progression. Furthermore, identifying these genes and understanding their functions has been critical in allowing us to better understand the pathogenesis of glaucoma in their identification of key biological signalling pathways, such as those involved in ER stress, which play a role in glaucoma development and progression. The timeline of milestone gene discoveries in glaucoma is shown in Figure 4.

### 5.2. Trabecular Meshwork

The TM is a key part of the aqueous humour outflow pathway of the eye. It is a complex tissue consisting of connective tissue beams and lamellae covered by TM cells [170]. In the TM, several studies have shown that the interplay between ECM accumulation and ER stress leads to increased outflow resistance and increased IOP [171,172]. In 2012, Suntharalingam showed that GRP94 depletion reduced mutant myocilin build up in human embryonic kidney by preferentially using autophagy rather than ERAD [149]. A large amount of our knowledge of ER stress in TM comes from the work of Zode et al., who in 2015, went on to look at glaucomatous TM (GTM) and normal TM (NTM), and showed that GTM cells had an increased GRP78 and GRP94 expression [173]. They also demonstrated that markers of chronic ER stress such as CHOP had a 3-fold increase in GTM, with ATF4 also being significantly enriched. This group, in 2017, showed that the increased ECM accumulation in the TM in glaucoma led to ER stress, in the TM more so than in other ocular tissues [174]. In 2018 they showed that in glaucoma induced by glucocorticoid use, increased extracellular matrix (ECM) deposition and ER stress were induced through TGFβ signalling, and interestingly, knockdown of ATF4 or CHOP completely prevented this signalling, thereby preventing ECM deposition and ER stress in mouse models of TM [175]. Most recently in 2020, Zode’s group showed that GTM cells had a significantly increased level of protein synthesis as well as induction of the chronic ER stress pathway ATF4-CHOP-GADD34, and demonstrated that in a mouse model, inhibition of this pathway prevented TM cell death and reduced protein synthesis and ER client protein load in TM cells [176]. Meanwhile, through in vitro studies of human TM cells and in vivo studies of mice TM, Ying et al. showed that ATF4 is a crucial mediator of both oxidative stress and ER stress-induced TM cell dysfunction and apoptosis [177].

### 5.3. Optic Nerve Head

The ONH is a structure in the posterior pole of the eye which allows the exit of retinal ganglion cell (RGC) axons and the entry and exit of blood vessels. This can be divided into three layers—the opening in Bruch’s membrane, the choroidal opening and the scleral opening [178]. This scleral opening is covered by the lamina cribrosa, a multi-layered network of load-bearing trabeculae that provide structural support to the optic nerve head and supply nutrients to the retinal ganglion cell axons as they leave the eye, as well as serving as a pressure barrier between the intravitreal and retrobulbar spaces [179]. Damage at the optic nerve head is the end result of glaucoma, and as such the optic nerve head is a key focus of glaucoma research. In 2012, Shimazawa et al. showed that RGC death led to an increase in GRP78, GRP 94 and CHOP, key markers of ER stress, in the optic nerve head 14 days later [180]. Ojino et al., in 2015 using the DBA/2J mouse, investigated the relationship between ER stress and the ONH in glaucoma and showed that ER stress may be related to astrocyte activation, playing a role in optic nerve degeneration in the setting of chronic ocular hypertension [181]. Stowell et al., using a primate model, demonstrated that several key ER chaperone proteins including GRP78, were significantly increased in the ONH in early experimental glaucoma [182]. In 2020, Mesentier-Louro et al. demonstrated that systemic hypoxia caused increased CHOP expression in astrocytes, and a loss of oligodendrocytes [183].

### 5.4. Retinal Ganglion Cells

RGCs are neurons which connect the retinal input to visual processing centres. Their axons converge at the optic disc and become myelinated, forming the optic nerve [184]. RGC loss is the hallmark of many optic neuropathies, including glaucoma, where loss occurs at the level of the optic nerve head [185]. While this review focuses on RGC and ER stress in glaucoma, there are many studies implicating ER stress in retinal diseases such as age-related macular degeneration, retinitis pigmentosa and Stargardt disease [186,187,188]. The ATF6 pathway has also been intensely studied in relation to RGC due to its genetic link to achromatopsia [189,190]. Hata et al., in 2008, induced retinal ischemia in rats, and showed that IRE1α was significantly increased in ganglion cells post ischaemia and subsequent reperfusion [191]. Doh et al., in 2010, in a rat model of glaucoma, tested the third ER stress pathway, the PERK pathway, and demonstrated a significant increase in GRP78 and CHOP expression, and a decrease in the number of ganglion cells compared to controls [192]. Hu et al. showed that inhibition of the PERK-eIF2α-CHOP pathway and activation of XBP1 can protect RGC soma and axons in mouse models of glaucoma, but in 2019, Marola et al. demonstrated that CHOP deletion alone, while conferring mild protection to RGCs, did not prevent axonal degeneration [193,194]. Yang et al., building on their previous work showing that optic nerve injury induced neuronal ER stress plays an important role in RGC death, used mouse models of both traumatic and glaucomatous optic neuropathies to show that manipulation of the UPR promotes RGC survival [195]. In 2021, Hetzer et al. demonstrated that in traumatic optic neuropathy, elevated ER stress markers were found in RGCs, as well as increased RGC death, astrogliosis and microgliosis [196].

## 6. Potential Therapeutic Options

### 6.1. Targeting Oxidative Stress

#### 6.1.1. Naturally Occurring Anti-Oxidants

Anti-oxidants, which scavenge and neutralise free radicals, offer a promising avenue for glaucoma therapeutics. Whilst anti-oxidants, such as glutathione and vitamin C, are found in high levels in the vitreous humour, their ability to buffer ROS is exceeded and oxidative stress occurs as a result [197,198]. Multiple naturally occurring anti-oxidants have shown encouraging results at a laboratory level that may replete this defence system. Maher et al. showed that flavonoids, low molecular weight phenol metabolites with anti-oxidative properties, prevented RGC-5 cell death in various oxidative stress conditions [199]. Flavonoids achieved this protective effect through the synthesis transcription factor NF-E2-related factor 2 (Nrf2) and antioxidant enzymes such as heme oxygenase 1 (HMOX1). Li et al. also found lutein, a carotenoid, to be protective against RGC-5 cell death induced by oxidative stress (H_2_O_2_) and hypoxia (cobalt chloride) [200]. Inman et al. examined the effects of an oral intake of α-lipoic acid (ALA)—a disulfide compound synthesised in the liver—in mouse models and found it improved RGC survival whilst reducing all markers of oxidative stress [201]. A study on 45 human POAG patients found that an oral anti-oxidant supplement, which included ginkgo biloba, significantly increased blood flow velocities in all retrobulbar blood vessels leading to improved ocular perfusion.

#### 6.1.2. Synthetic Anti-Oxidants

Exogenous anti-oxidative therapies also may offer protective effects against glaucomatous neurodegeneration. Rapamycin, an mTOR inhibitor which alleviates oxidative stress, has been shown to suppress apoptosis of RGCs in rats with chronic ocular hypertension [202]. It also been found to have a protective effect on TM cells in steroid-induced glaucoma in mouse models [203].

Metformin, a commonly used medication for type 2 diabetes mellitus, has emerged as a drug of interest in combating the fibrotic changes and mitochondrial dysfunction seen in glaucomatous eyes. In a large 150,016 patient retrospective cohort study in the United States of patients with type 2 diabetes mellitus (T2DM), aged ≥ 40 and with no pre-existing POAG, Li et al. found up to a 25% reduced risk of POAG amongst diabetics taking a high dose of metformin (>1110 g in 2 years) compared to those who took no metformin after adjusting for confounding factors [204]. It has potent anti-oxidative effects, increasing anti-oxidant capacity [205,206] and decreasing ROS production through the inhibition of protein kinase C activity [207,208].

Netarsudil is a Rho kinase (ROCK) inhibitor with anti-oxidative effects that has been approved for glaucoma treatment [209]. A similar ROCK inhibitor, Y-26732, has been shown to downregulate ROS production and upregulate anti-oxidants such as catalase in cynomolgus monkey TM cells [210]. Other anti-oxidants that have been shown to be protective in lab-based glaucoma studies include nicotinamide (vitamin B3) [211], N-acetylcysteine [212] and edaravone [213].

The systemic administration of medications may offer good local protection as the redox status of ocular tissues closely mirrors that of systemic capacity. Intravitreal injections of antioxidants, resveratrol [214] and ubiquinone (Coenzyme Q10) [215], have shown excellent results in alleviating RGC damage in murine models but have not been tested in a human population.

### 6.2. Targeting Endoplasmic Reticulum Stress

#### 6.2.1. 4-Phenylbutyric Acid (4-PBA)

The most widely studied novel potential treatment of ER stress in glaucoma is 4-PBA. Initially, 4-PBA was used as a treatment for urea cycle disorders in the early 1990s [216]. In 1996, it was identified as a potential treatment for cystic fibrosis, through its action as a chemical chaperone [217]. Roth et al. hypothesised in 2007, that as myocilin- associated glaucoma is essentially a protein misfolding disease, treatment with a chemical chaperone represented a novel therapeutic option [218]. They demonstrated that the treatment of human TM cells (that had been previously transfected to express mutant myocilin) with 4-PBA significantly relieved ER stress and decreased the rate of apoptosis, while the treatment of TM cells with other chemical chaperones such as glycerol, did not. In 2011, Zode et al. demonstrated that 4-PBA treatment of mice (through the addition of 4-PBA to drinking water) with myocilin mutations, decreased ER stress and prevented glaucoma phenotypes [219]. They went on, in 2012, to prove that topical application of 4-PBA eyedrops also prevented the development of glaucoma phenotypes [220]. In 2021, Zode et al. showed that the benefit of 4-PBA is not restricted to its role as a chemical chaperone alone, but that it also prevents the synthesis and deposition of glucocorticoid induced ECM in TM, as well as degrading existing abnormal ECM by inducing matrix metalloproteinase 9 expression and activity [221]. In 2019, Kumar et al. showed that treatment with 4-PBA post non-arteritic anterior ischaemic optic neuropathy rescued RGCs and oligodendrocytes, and in 2020, Mesentier-Louro et al. showed that the pre-treatment of mice with 4 -PBA pre systemic hypoxia significantly reduced ER stress and rescued mature oligodendrocytes [183,222]. Given its previous approval for oral use for the treatment of urea cycle disorders and good safety profile, 4-PBA is an exciting potential glaucoma treatment.

#### 6.2.2. Salubrinal

First identified by Boyce et al. in 2005, in a screen for small molecules that protect cells from ER stress, salubrinal is a selective inhibitor of cellular complexes that dephosphorylate eIF2α [223]. This means the PERK pathway remains active. Tested in human TM cells by Wang et al. in 2019, salubrinal was shown to be protective against cell death [224].

#### 6.2.3. CRISPR-Cas9

CRISPR-Cas9 is a unique and exciting technology that enables editing of the genome through altering sections of the DNA sequence [225]. The discovery that CRISPR-Cas 9 could be programmed with RNA to edit genomic DNA won Doudna and Charpentier the Nobel Prize in Chemistry in 2020 [226]. We have already discussed the many genes that have been linked to glaucoma, and in particular, MYOC. In 2017, Sheffield et al. used CRISPR-Cas9 to knock down the expression of mutant MYOC in an in vivo mouse model, showing relief of ER stress, reduction in IOP and prevention of further glaucomatous damage [227]. They also showed its utility in human cultured TM cells, as well as ex vivo perfusion cultured human eyes. Wu et al., in 2020, used a mouse model and ex vivo human ciliary body model to demonstrate the efficacy of the CRISPR-Cas9 system in disrupting the Aquaporin 1 gene to treat glaucoma, with no off target changes to the retina or cornea identified [228].

## 7. Conclusions

**Figure 5:**
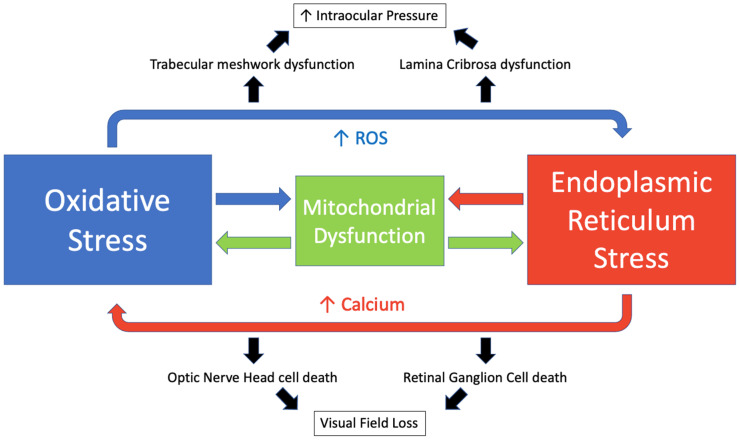
The interplay of oxidative stress and endoplasmic reticulum stress in glaucoma.

Oxidative and endoplasmic reticulum stress are key drivers of glaucoma, and their mechanisms act in an interconnected manner with calcium (Ca^2+^) acting as a key intermediary. Ca^2+^ and ROS act in a reciprocal manner. Increased calcium levels activate the ROS-generating enzymes and formation of free radicals [229]. Meanwhile, increased ROS stimulates an increase in intracellular calcium concentration [230]. The endoplasmic reticulum is the main intracellular calcium store with approximately 2 mM total Ca^2+^ [230]. During states of ER stress, calcium is released via inositol 1,4,5-trisphosphate (IP3) receptors and ryanodine receptors and accumulates in the mitochondrial matrix [231]. This promotes mitochondrial permeability transition pore opening, ROS accumulation and ATP depletion [232]. ROS may then disrupt the protein folding mechanism, which is dependent on redox homeostasis [21]. This perpetuates a cycle resulting in a further increase in oxidative and ER stress, mitochondrial dysfunction and the induction of apoptosis [233]. We have illustrated this cycle in Figure 5.

Approximately 25% of ROS generated in the cell are derived from the ER [234]. An increase in ROS may also lead to activation of the UPR [235]. ATF4, which promotes aberrant protein synthesis, has been linked to oxidative stress in several studies [236,237]. Kasetti et al. have shown that induced over-expression of ATF4 leads to increased ROS expression in human TM cells [176], while a study by Kuk Joe et al. found that the expression of myocilin mutants in human TM cells resulted in ER stress which sensitised cells to oxidative stress-induced apoptosis [238]. This interplay has also been described in multiple neurodegenerative diseases [239], cancer [240,241], diabetes mellitus [242], cardiovascular disease [243] and other diseases.

The evidence of oxidative and ER stress in glaucoma, at multiple sites, represents a new avenue of therapeutic potential. Given the cyclical nature of the processes, both may be targeted by one agent as evidenced by a study showing that an overexpression of the anti-oxidant superoxide dismutase resulted in attenuated ER stress and subsequent prevention of neuronal damage in ischaemic conditions [244]. The use of such therapies that target both pathways may allow clinicians to treat patients with normal tension glaucoma or those failing to respond to conventional pressure-lowering therapies. Glaucoma is a multifactorial disease process, in which oxidative stress and endoplasmic reticulum stress play a key role, thus, there is a clear unmet clinical need to address a disease modifying agent and this requires tackling a mechanism beyond IOP regulation.
